# Insight into the hepatoprotective, hypolipidemic, and antidiabetic impacts of aliskiren in streptozotocin-induced diabetic liver disease in mice

**DOI:** 10.1186/s13098-022-00935-5

**Published:** 2022-10-31

**Authors:** Amal M. Mahfoz, Aya Y. Gawish

**Affiliations:** Department of Pharmacology and Toxicology, Faculty of Pharmacy, Modern University for Technology and Information, Cairo, Egypt

**Keywords:** Aliskiren, Anti-inflammatory, Diabetes mellitus, Liver toxicity, Oxidative stress, Streptozotocin

## Abstract

**Background:**

Diabetic hepatopathy is a serious complication of poorly controlled diabetes mellitus. An efficient antidiabetic drug which keeps normal liver tissues is not available. The renin-angiotensin system has been reported to be involved in both diabetic state and liver function. Aliskiren is a direct renin inhibitor and a recently antihypertensive drug with poly-pharmacological properties. The aim of the current study is to explore the possible hepatoprotective effects and mechanisms of action of aliskiren against streptozotocin (STZ) induced liver toxicity.

**Methods:**

Mice were distributed to 3 groups; first: the normal control group, second: the diabetic control group, third: the diabetic group which received aliskiren (25 mg/kg; oral) for 4 weeks. At the end of the treatment period, plasma glucose, insulin, lipid profile, oxidative stress, and liver function tests were evaluated spectrophotometrically. ELISA technique was used to measure the expression levels of TNF-α and adiponectin. Furthermore, a Histopathological examination of liver samples was done.

**Results:**

It was shown that aliskiren treatment ameliorated the STZ-induced oxidative stress and elevated inflammatory biomarkers, hypercholesterolemia, serum aminotransferases and alkaline phosphatase levels in diabetic mice. In addition, hepatocellular necrosis, and fibrosis were improved by aliskiren treatment.

**Conclusion:**

aliskiren protects against the liver damage caused by STZ-induced diabetes. This can be explained by its ability to block angiotensin-II, and its anti-diabetic, hypocholesterolemic, antioxidant and anti-inflammatory effects. Aliskiren could be a novel therapeutic strategy to prevent liver diseases associated with hypertension and diabetes mellitus.

**Graphical Abstract:**

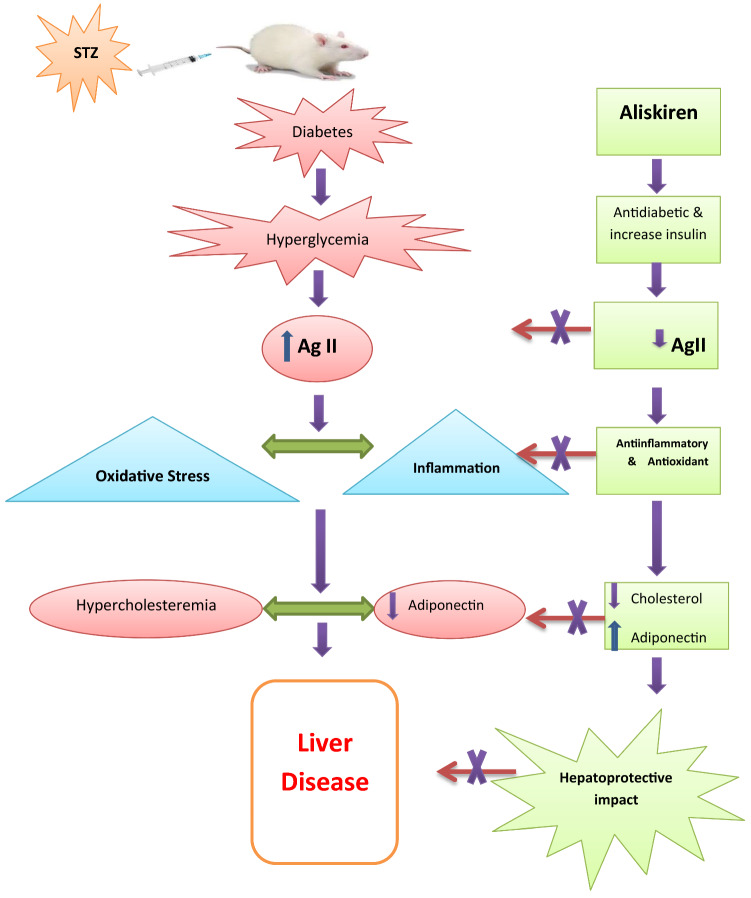

## Background

Diabetes mellitus (DM) is a chronic severe metabolic endocrine disorder. It is considered one of the most common and serious chronic disorders worldwide [1, 2]. Previous studies have reported that diabetic patients with poorer glycemic control suffer hepatomegaly, elevated levels of serum alanine transaminase (ALT) and aspartate transaminase (AST) [3, 4], high oxidative stress [5], hepatocellular necrosis and fibrosis [6]**.** Thus indicates liver impairment associated with DM [7, 8]**.**

Blocking of the renin angiotensin aldosterone system (RAAS) by different ways has reported to be involved in hepatic protective impacts [9–11]**.** Aliskiren (C30H53N3O6, Molecular weight: 551.8 g/mol) is the first approved drug in the recent antihypertensive class; direct renin inhibitors [12]**.** Aliskiren acts by down-regulation of the RAAS, lower plasma renin activity, plasma angiotensin I (Ang I), and angiotensin II (Ang II) [13, 14]**.** Ang II is produced through endothelial cleavage of Ang I. Ang I is produced from angiotensinogen by hepatocytes. Renin cleaves angiotensinogen to form Ang I which is then converted by ACE to the active Ang II [15]**.** Furthermore, the antidiabetic effects of aliskiren have been proven previously [16]. A previous study has reported that aliskiren reduced portal pressure and intrahepatic resistance in cirrhotic rat liver [17]**.** The current study aimed to estimate the hepatoprotective impacts and possible mechanisms of action of aliskiren in an experimental model of induced diabetes in mice using STZ.

## Methods

### Animals and experimental design

Male Swiss albino mice (25–30 g) were maintained at 20–25 °C in a 12 h light/dark cycle, with a commercial normal rodent diet and water freely available. They were divided into three groups, 8 mice each: 1st; normal control group (received citrate buffer, IP for 1 month), 2nd; diabetic control group (STZ-induced diabetic mice and received normal saline, IP, for 1 month). 3rd; diabetic mice were treated with aliskiren; three days after STZ injection, this group of mice received daily aliskiren (25 mg /kg oral, dissolved in sterile normal saline) for 1 month. The volume of injection was 0.25 ml/20 g mice. Different dose levels of aliskiren have been tested before in experimental studies [18, 19]**.** We have selected the least effective dose correlated to the clinical dose used in patients [20]. We did not try different dose levels to reduce the number of used animals according to the standard research ethics.

### Diabetes induction

Mice for the second and third groups were overnight fasted and then injected intraperitoneally with a single dose of 50 mg/kg streptozotocin dissolved in a freshly prepared citrate buffer (pH 4.5), 15 min after nicotinamide (NA) 110 mg/kg (dissolved in normal saline), to partially prevents the harmful effects of STZ to develop a model of non-insulin-dependent DM. Three days later; blood glucose was checked by a one-touch Glucometer, using blood from the tail. Mice with hyperglycemia (≥ 250 mg/dl) were selected as diabetic [21].

At the end of the treatment period, mice were fasted overnight. Blood samples were collected into tubes containing EDTA. Plasma was separated by centrifugation (10,000 rpm/min, for 10 min, 4 °C). Liver samples were separated, washed with saline, and kept in 10% formalin.

STZ, aliskiren, and ELISA kits were purchased from Sigma Aldrich Co (St Louis, MO, USA). Other chemicals and reagents were of analytical grade and were supplied by Biodiagnostic company, Egypt.

### Biochemical analysis

#### Blood glucose and serum insulin

Fasting blood glucose was assessed spectrophotometrically using blood from the lateral tail vein on the last day of the experiment; by a commercial kit (Biodiagnostic, Egypt) [22]. Serum insulin was assessed using the ELISA technique.

#### Oxidative stress biomarkers

Serum oxidative stress biomarkers and antioxidant enzymes [reduced Glutathione (GSH), Superoxide dismutase (SOD), Malondialdehyde (MDA), and Nitric oxide (NO)] were measured spectrophotometrically using commercial reagent kits. SOD was assessed in line with the pyrogallol autoxidation technique [23]. MDA was estimated according to Satoh [24]. GSH was measured in agreement with Beutler et al*.* [25].

#### Liver functions markers and lipid profile

Serum albumin level was assessed in agreement with the modified bromocresol green technique [26]. Serum ALT and AST were assayed according to Piyachaturawat et al. [27].

The level of serum alkaline phosphatase was assayed according to Reitman and Frankel [28]. CPK-total was assayed according to Bishop et al. [29]. Cholesterol and triglyceride were determined enzymatically using a commercial kit according to Cox and García-Palmieri [30].

#### Adiponectin

Adiponectin was quantitatively measured in serum using a commercial ELISA kit (Biovision, Inc., San Francisco). The principle of the assay is that polyclonal antibody specific for adiponectin has been pre-coated into 96 well microplate. Standards and samples were pipetted into the wells and any adiponectin present is bound by immobilized antibody. The bound adiponectin is then captured by anti-adiponectin monoclonal antibody. With adding HRP conjugated anti-mouse IgG and HRP substrate, the colors developed in proportion to the bound adiponectin, can be easily measured by Elisa plate reader [31]**.**

#### Tumor necrosis factor-alpha (TNF-α)

TNF-α was measured using BioVision’s ELISA Kit. This assay employs a monoclonal antibody specific for mouse TNF-α coated on a 96-well plate [32]**.**

### Histological preparation

Liver samples were flushed and fixed in 10% neutral buffered formalin for 72 h. Samples were trimmed and processed in serial grades of ethanol, cleared in Xylene, synthetic wax infiltration and embedding into Paraplast tissue embedding media. 5μn tissue sections were cut by rotatory microtome then fixed into glass slides and stained by Hematoxylin and Eosin (H and E) stain. Then examined by experienced histologist in blinded manner by using Full HD microscopic imaging system “(Leica Microsystems GmbH, Wetzlar, Germany)”. All standard procedures for samples fixation, processing and staining were done according to Culling, C.F.A. [33].

### Statistical analysis

The data were obtained from at least 3 different experiments and were expressed as means ± S.E. Results were calculated by one-way ANOVA, followed by Tukey’s test as a post hoc test. P < 0.05 was considered significant. All the analyses were carried out by using SPSS software (version 22.0) for Windows 8.1 (SPSS, Inc., Chicago, IL, USA).

## Results

### Blood glucose and serum insulin level

STZ leads to significant hyperglycemia associated with a significant decrease in serum insulin. Treatment with aliskiren leads to a significant lowering in blood glucose and increase in serum insulin as related to STZ only treated group, P < 0.05 (Table 1).

**Table 1:** Effect of one month treatment with aliskiren on plasma glucose, oxidative stress markers, and antioxidant enzymes in STZ-induced diabetes in mice

Parameter Group	Blood glucose (mg/dl)	Insulin (μIU/ml)	GSH (mg/dl)	SOD (U/ml)	MDA (nmol/ml)	NO (µmol/µl)
Normal control	105.83 ± 4	67.67 ± 1.5	9.4 ± 0.67	96.17 ± 1.38	25.15 ± 0.27	87.16 ± 2.41
Diabetic control	448.17 ± 66.24^a^	26.17 ± 0.7^a^	4.85 ± 0.06^a^	61.1 ± 0.7^a^	40.97 ± 0.39^a^	240 ± 2.11^a^
Aliskiren	161 ± 15.4^a,b^	64.17 ± 1.1^b^	6.59 ± 0.11^a,b^	100.17 ± 0.57^a,b^	27.34 ± 0.32	140.67 ± 0.88^a,b^

Each value represents mean of 8 mice ± SEM. Statistical analysis was carried out using one way analysis of variance (ANOVA) followed by Tukey Kramer multiple comparisons test

^a^Significantly different from Normal control at P < 0.05

^b^Significantly different from Diabetic control at P < 0.05

### Oxidative stress markers

STZ treated group showed a significant elevation in oxidative stress biomarkers (MDA and NO). This was accompanied by a significant reduction in plasma antioxidants (GSH, SOD). Treatment with aliskiren demonstrated a significant reduction of oxidative stress markers (MDA and NO) and significant elevation in GSH and SOD, P < 0.05 (Table 1).

### Liver functions markers and Lipid profile

**Fig. 1:**
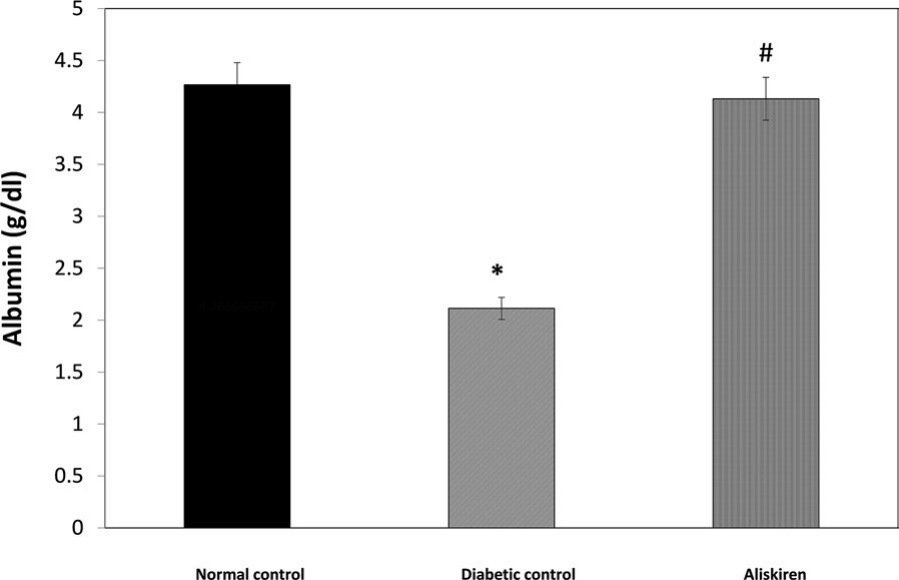
Effect of one month treatment with aliskiren on Albumin level in STZ-induced diabetes in mice. Each value represents mean of 8 mice. Statistical analysis was carried out using one way analysis of variance (ANOVA) followed by Tukey Kramer multiple comparisons test. ^*^Significantly different from Normal control at P < 0.05. ^#^Significantly different from Diabetic control at P < 0.05

**Fig. 2:**
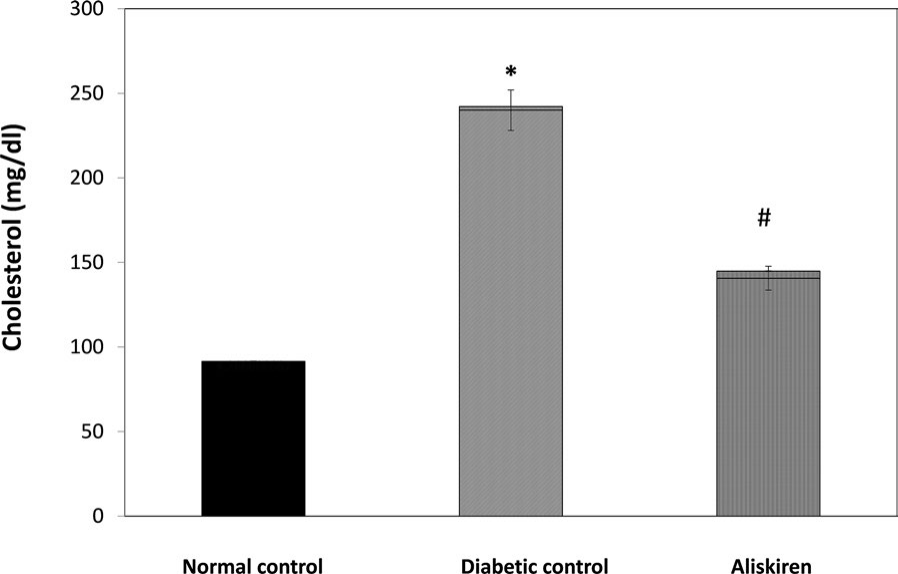
Effect of one month treatment with aliskiren on Cholesterol level in STZ-induced diabetes in mice. Each value represents mean of 8 mice. Statistical analysis was carried out using one way analysis of variance (ANOVA) followed by Tukey Kramer multiple comparisons test. ^*^Significantly different from Normal control at P < 0.05. ^#^Significantly different from Diabetic control at P < 0.05

**Fig. 3:**
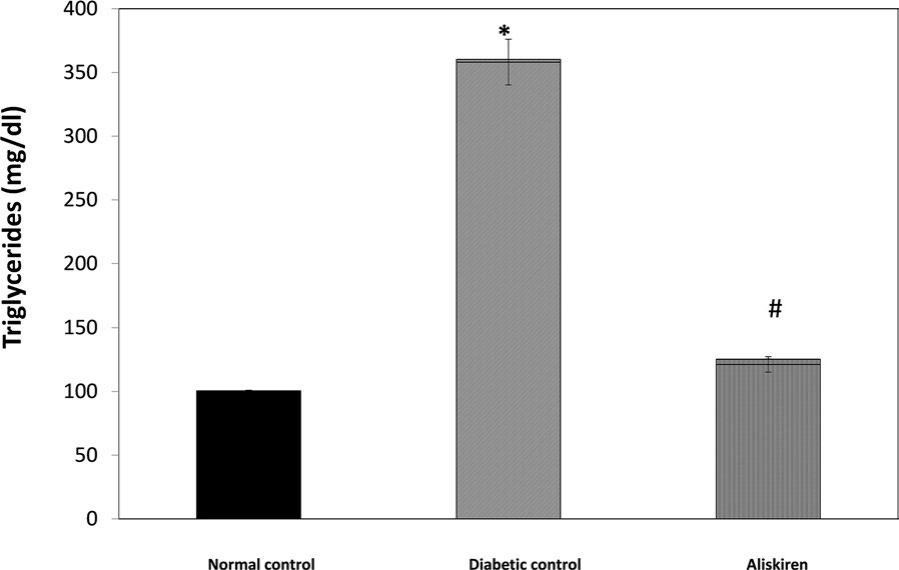
Effect of one month treatment with aliskiren on Triglyceride in STZ-induced diabetes in mice. Each value represents mean of 8 mice. Statistical analysis was carried out using one way analysis of variance (ANOVA) followed by Tukey Kramer multiple comparisons test. ^*^Significantly different from Normal control at P < 0.05. ^#^Significantly different from Diabetic control at P < 0.05

STZ resulted in impairment of liver function markers that was demonstrated by the significant elevation in AST, ALT, ALP, and CPK-total. This was accompanied by a significant reduction in albumin content. In addition to a significant elevation in lipid profile as demonstrated by the significant increase in TG and C, P < 0.05 (Figs. 1, 2, 3, Table 2).

**Table 2:** Effect of one month treatment with aliskiren on liver function markers in STZ-induced diabetes in mice

Parameter Group	AST (IU/L)	ALT(IU/L)	ALP(IU/L)	CPK-total
Normal control	35.33 ± 1.48	40.33 ± 0.76	87.16 ± 1.96	150 ± 1.15
Diabetic control	316.5 ± 1.38^a^	433.33 ± 14.15^a^	350 ± 1.81^a^	849.83 ± 270.31^a^
Aliskiren	42.72 ± 0.52^a,b^	56.83 ± 1.45^b^	90.03 ± 0.58^b^	188.33 ± 0.88^b^

Each value represents mean of 8 mice ± SEM. Statistical analysis was carried out using one way analysis of variance (ANOVA) followed by Tukey Kramer multiple comparisons test

^a^Significantly different from Normal control at P < 0.05

^b^Significantly different from Diabetic control at P < 0.05

Treatment of the diabetic group with aliskiren for 1 month resulted in a significant reduction of TG and C and liver function tests (AST, ALT, ALP, CPK-total). In addition to, a significant increase in plasma albumin content, P < 0.05 (Figs. 1, 2, 3, Table 2).

### Adiponectin level

**Fig. 4:**
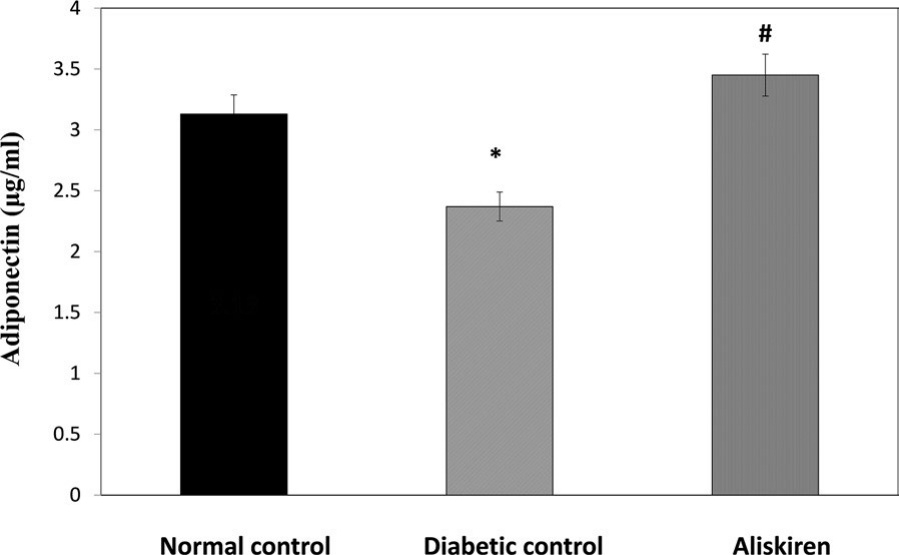
Effect of one month treatment with aliskiren on adiponectin level in STZ-induced diabetes in mice. Each value represents mean of 8 mice. Statistical analysis was carried out using one way analysis of variance (ANOVA) followed by Tukey Kramer multiple comparisons test. ^*^Significantly different from Normal control at P < 0.05. ^#^Significantly different from Diabetic control at P < 0.05

A significant reduction in serum adiponectin was observed in the diabetic group. However, a significant increase in serum adiponectin was observed in aliskiren treated group (Fig. 4).

### Tumor necrosis factor-α (TNF-α)

**Fig. 5:**
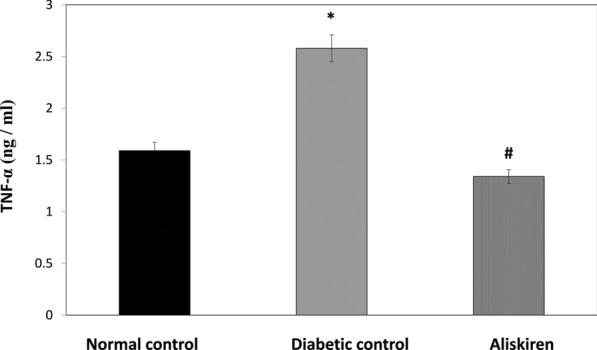
Effect of one month treatment with aliskiren on Tumor necrosis factor—alpha in STZ-induced diabetes in mice. Each value represents mean of 8 mice. Statistical analysis was carried out using one way analysis of variance (ANOVA) followed by Tukey Kramer multiple comparisons test. ^*^Significantly different from Normal control at P < 0.05. ^#^Significantly different from Diabetic control at P < 0.05

The diabetic control group showed a significant elevation in serum TNF-α. Aliskiren treatment leads to a significant reduction in TNF-α in comparison to STZ diabetic mice (Fig. 5).

### Histological examination

**Fig. 6:**
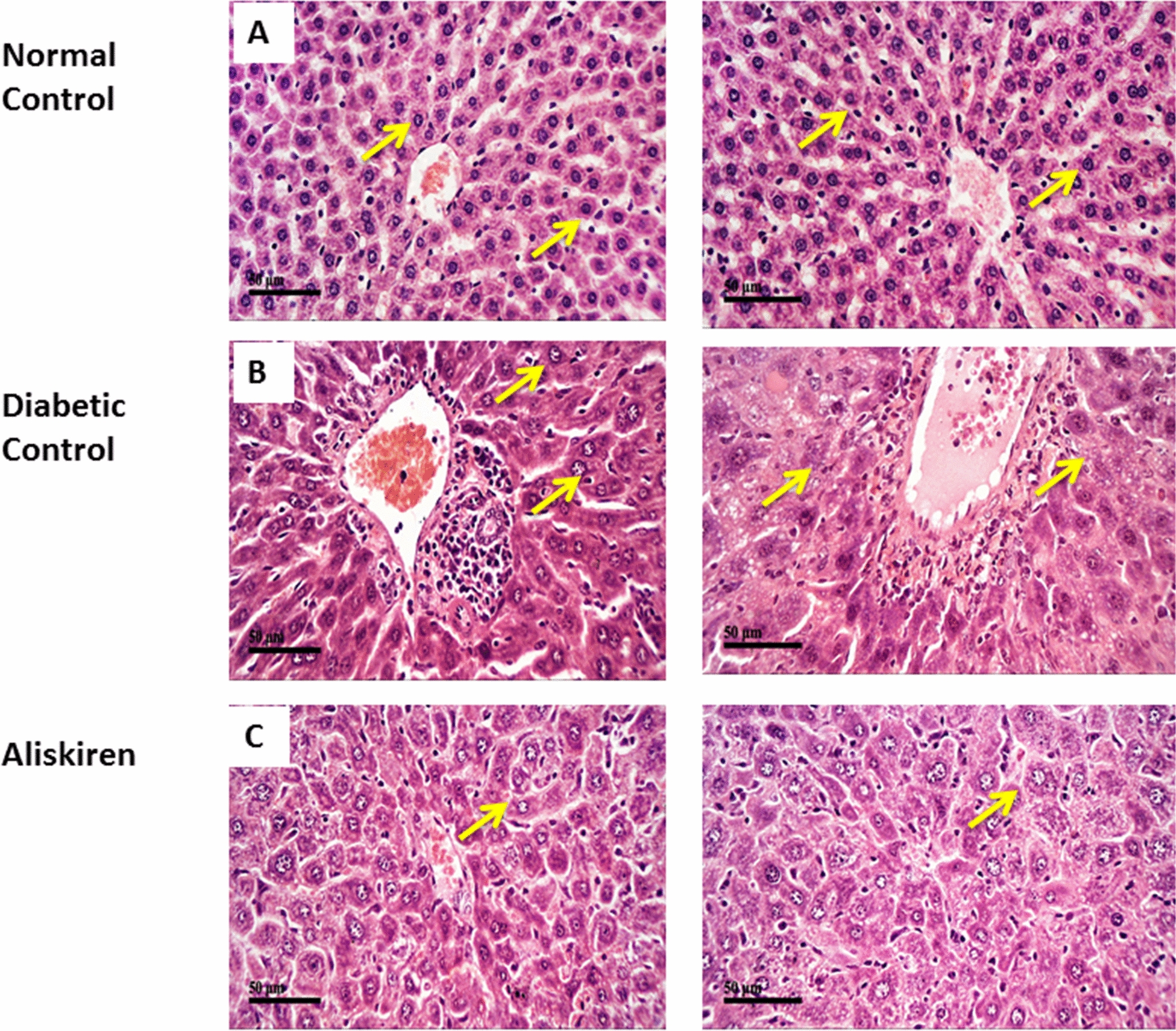
Effect of one month treatment with aliskiren on Microscopic examination of different hepatic tissue sections in STZ-induced diabetes in mice (H and E-stained) (× 400). a: normal control, b: diabetic control and c: aliskiren treated group. **a** Normal controls demonstrated normal morphological features of hepatic parenchyma with many apparent intact hepatocytes having large vesicular nuclei***, ***intact hepatic vasculatures were observed with minimal degenerative changes. **b** Diabetic model samples showed significant periportal and perivascular inflammatory cells infiltrates with mild vacuolar degenerative changes of hepatocytes with many nucleocytomegaly records with prominent nucleoli*.* **c** Aliskiren treated group showed minimal inflammatory cells infiltrates records with more apparent intact hepatocytes with occasional nucleocytomegaly and or binucleation alternated with fewer degenerated cells accompanied with activated kupffer cells

*Microscopic examination of different hepatic tissue sections showed; normal controls* demonstrated normal morphological features of hepatic parenchyma with many apparent intact hepatocytes having large vesicular nuclei*,* and intact hepatic vasculatures were observed with minimal degenerative changes. *Diabetic model samples* showed significant periportal and perivascular inflammatory cell infiltrates with mild vacuolar degenerative changes of hepatocytes with many nucleocytomegaly records with prominent nucleoli*.* Aliskiren treated group showed minimal inflammatory cell infiltrates records with more apparent intact hepatocytes with occasional nucleocytomegaly and or binucleation alternated with fewer degenerated cells accompanied with activated kupffer cells (Fig. 6).

## Discussion

Diabetes Mellitus (DM) is a common endocrine and metabolic disorder. It is considered a major health care threat worldwide due to its severe complications. Streptozotocin (STZ); a pancreatic cytotoxic induces irreversible necrosis of cells. It is used in the induction of diabetes experimentally as it mimics the endogenous chronic tissue damage and oxidative stress as a result of hyperglycemia [34–36]. The liver is the major organ of glucose metabolism in response to insulin. Liver is responsible for detoxification, clearance of oxidative stress bioproducts, regulating glycolysis, and gluconeogenesis [37, 38]. Long term hyperglycemia in poorly controlled DM may lead to liver disease. Moreover, liver impairment can predispose prediabetes or type 2 DM [7].

Diabetic mice, in the current study, had demonstrated significant hyperglycemia associated with a decrease in insulin level. Aliskiren treatment interrupted the observed increase in glucose level and decrease in insulin when compared to the diabetic group. This result is supported by previous studies demonstrating that aliskiren stimulates insulin secretion in-vitro from isolated β-cells and decreases insulin resistance in-vivo [16]. The decreased plasma glucose after aliskiren treatment can be explained by its ability to increase insulin secretion or enhance insulin sensitivity [39]. Aliskiren also upregulates glucose transporters expression levels in the liver (GLUT 2) and muscle (GLUT 4), these confirm the improvement of insulin resistance by aliskiren [40, 41].

Furthermore, aliskiren increases the plasma level of adiponectin. Adiponectin is a novel adipocytokine produced mainly in adipose tissue and is involved in regulating glucose levels, fatty acid breakdown, and insulin metabolism. It plays a role in the suppression of metabolic abnormalities that may result in type 2 diabetes. Adiponectin therapy has been shown to induce beneficial metabolic effects in animals by decreasing both hepatic gluconeogenesis and plasma triglyceride levels and has also antiatherogenic and anti-inflammatory effects [42]. Adiponectin enhances insulin sensitivity primarily through upregulation of fatty acid oxidation and suppression of hepatic glucose production [43, 44]. So adiponectin stimulation by aliskiren leads to increasing insulin sensitivity. In addition to, its insulin stimulation and hypoglycemic properties.

In line, high oxidative stress is involved in the bad prognosis of DM and worsens its complications on the liver [45, 46]. A possible explanation may be that reactive oxygen species (ROS) could interact with proteins, lipids, and DNA, resulting in the dysfunction of these important macromolecules [47]. ROS generates oxidative stress, which accelerates the damage and destruction of many organs [48, 49]. So, antioxidant enzymes including GSH, and SOD have potential protective impacts against tissue damage by their ability to decompose ROS and block lipid peroxidation [49, 50].

Lipid peroxidation is induced by the interaction of ROSand polyunsaturated fatty acids and results in the formation of MDA, which represents cellular damage and cytotoxicity [50]. Evidence has reported that high lipid peroxidation leads to the progression of DM by altering the normal functions of membrane-bound enzymes and receptors. Oxidative stress leads to the development of microvascular and cardiovascular diabetic complications. Hyperglycemia causes mitochondrial superoxide overproduction in endothelial cells of both large and small vessels. This increased superoxide production causes the activation of five major pathways involved in the pathogenesis of diabetes complications: polyol pathway flux, increased formation of advanced glycation end-products (AGEs), increased expression of the receptor for AGEs and its activating ligands, activation of protein kinase C isoforms, and overactivity of the hexosamine pathway. It also directly inactivates two critical antiatherosclerotic enzymes, eNOS and prostacyclin synthase [51].

In the current study, we observed a significant reduction in the activities of SOD, and GSH and an increase in MDA, and NO serum levels in STZ-induced diabetic mice. However, a significant elevation in antioxidants and a remarkable lowering in lipid peroxidation were observed in the aliskiren treated group. This was indicated by the elevation of SOD, GSH, and the reduction in MDA, NO. The antioxidant potential of aliskiren was in line with previous studies. Ang II may possess a role in phosphorylation and rise of ROS in the liver [52] and contributed to the bad prognosis of non-alcoholic fatty liver disease by elevating hepatic ROS [53]. Oxidative stress also triggers the development of steatohepatitis by stimulating inflammatory response [54]. So, the blockade of Ang II by aliskiren resulted in its antioxidant potential which also contributes to its potency to decrease insulin resistance and protects hepatocytes from high oxidative stress induced by DM.

In addition, hepatic ROS, as well as proinflammatory cytokines (TNF-α, IL-1β, and IL-6) are reduced with Ang-II inhibition [55]**.** As observed in the current study that aliskiren ameliorated the STZ-induced elevation in TNF-α. Aliskiren possesses anti-inflammatory properties leads to the preservation of liver function markers.

Increased activities of serum aminotransferases are also diagnostic markers of liver disorder and are occurred more frequently in diabetic patients [56, 57]. The present study demonstrated that serum ALT and AST levels were significantly elevated in STZ-treated animals, these were following previous studies [58–60] and indicated hepatocellular necrosis [61]. It has also been reported that high serum ALT and AST are involved in increasing insulin resistance and defective utilization of glucose by the liver [62]. Insulin deficiency leads to the breakdown of protein and enhances amino acid catabolism to provide substrates for gluconeogenesis [63].

Administration of aliskiren significantly lowered AST and ALT activities, indicating that it had potential effects on improving liver function. These results agreed with those of Hsieh et al. [17] who demonstrated that there is a beneficial effect of aliskiren on portal pressure and intrahepatic resistance through reduction of Ang II production in the cirrhotic liver. These authors concluded that direct renin inhibition may serve as a potential and effective therapeutic strategy for the management of portal hypertension.

A high level of ALP was demonstrated in the STZ diabetic group. Increased ALP has been reported in pathological conditions which involve the kidney and liver [64, 65]**.** In addition, diabetic mice showed serious lipid dysfunction, which was experimentally validated by the increased levels of TG and TC similar to the clinical properties of human DM [66]. TC and TG are important parameters for evaluating blood viscosity, and the risk of atherosclerosis. The administration of aliskiren significantly decreased TG and TC, this indicated its potential impacts on improving lipid metabolism and was in accordance with a previous study that demonstrated increased hepatic turnover of triglycerides with an upregulation in fatty acid transport and breakdown after aliskiren treatment [9]**.** So, the RAAS influences hepatic fatty acid metabolism.

Finally, the microscopic examination of liver tissue from STZ-treated mice revealed loss of hepatic architecture, hepatomegaly, dilatation of hepatic sinusoid capillaries close to the central vein, apoptotic hepatocytes, and hepatocytes with lipid droplets in their cytoplasm indicating increased adipogenesis and cell death as well as signs of inflammation, which were all mitigated by aliskiren treatment [58, 67]**.**

From the shown data, liver fibrosis and its serious complications such as portal hypertension and hepatocellular carcinoma are evidenced to be related to the bad prognosis of diabetes [68, 69]**.** Plasma renin activity and angiotensins were reported to be increased in advanced liver disease [70–72] which indicates the role of RAAS in diabetes-induced liver impairment. So, inhibition of Ang II synthesis by aliskiren may attenuate hepatic fibrosis as observed in the current and previous studies [73, 74]**.** Aliskiren has been proven as an effective way to block renin activity and restrain liver fibrosis in experimental models. In addition to its explained hepatoprotective mechanisms by lowering the induced high blood glucose, lipid profile, liver enzymes, oxidative stress, and inflammatory biomarkers.

## Conclusion

Aliskiren is a direct renin inhibitor, that prevents the formation of Ang II by blocking renin from converting to Ang I. Our study is the first to demonstrate the hepatoprotective impacts and mechanisms of aliskiren in a model of STZ-induced diabetes in mice. The evidenced hepatoprotective effect was a result of improvement of RAAS by inhibition of Ang II production, Ang II action, glycemic control, reduction of insulin resistance, changes in lipid metabolism, anti-inflammatory and antioxidant effects elicited by aliskiren treatment. Aliskiren is considered a promising treatment for the underlying conditions associated with hypertension, hypercholesterolemia, and diabetes. Clinical studies are essential to ensure its impact on the management of hypertensive or diabetic patients with liver diseases.

## Data Availability

The datasets used and/or analyzed during the current study are available from the corresponding author upon reasonable request.

## Abbreviations

AGEs: Advanced glycation end-products
ALT: Alanine transaminase
ALP: Alkaline phosphatase
ACE: Angiotensin converting enzyme
Ang I: Angiotensin I
Ang II: Angiotensin II
AST: Aspartate transaminase
CPK-total: Creatine phosphokinase
DM: Diabetes mellitus
eNOS: Endothelial nitric oxide synthase
H and E: Hematoxylin–eosin
IL-1β: Interleukin-1β
IP: Intraperitoneal
MDA: Malondialdehyde
NO: Nitric oxide
NF-κB: Nuclear factor kappa B
GSH: Reduced glutathione
RAAS: Renin angiotensin aldosterone system
ROS: Reactive oxygen species
STZ: Streptozotocin
SOD: Superoxide dismutase
TNF- α: Tumor necrosis factor-alpha
TC: Total cholesterol
TG: Triglyceride
